# Supplementary material for: The Health of Healthcare Professionals in Italian Oncology: An Analysis of Narrations through the M.A.D.I.T. Methodology
Source: Behav Sci (Basel). 2022 May 5;12(5):134. doi: 10.3390/bs12050134 (PMC9137842; doi:10.3390/bs12050134)
Supplement: Supplementary file 1 [file behavsci-12-00134-s001.zip › behavsci-1620799-supplementary.pdf]

Table S1. Investigation Questionnaire.

Area's aim	Question	Target categories
A. How would you describe the configuration of "Healthcare professional who follows cancer patients" enunciated as etero-attribution, self-attribution, criticalities and strengths?	1. Imagine you find a small notebook, you open it, you read a few lines and affirm: "This must be the private diary of a person who takes care of cancer patients". Which are the sentences that you find there in order to say so? (Etero-attribution)	All categories
	2. Describe 2 critical aspects that the healthcare professional already cited could encounter in relation with his/her role.	OPSUN, "Nucleo affettivo" e pazienti oncologici
	3. How do you think these critical aspects could be managed?	OPSUN
	4. Describe 2 strengths that the same professional could define as such compared to his/her role	OPSUN, "Nucleo affettivo" e pazienti oncologici
	6. a) Imagine that a publishing house asks you to write a book on your life as doctor/ nurse/ social-health worker working with cancer patients. How would you describe your typical day during the shift? (Self-attribution)	OPSUN
	6. b) Imagine to be a doctor/nurse/social- health worker who works with cancer patients and that a publishing house asks you to write a book on your story. How would you describe your typical day during your shift? (Self-attribution)	"Nucleo affettivo", pazienti oncologici e altri cittadini
	7. How would you describe your spare time? (Self-attribution)	All categories
	8. How would you describe the changes happened because of the pandemic situation caused by the SARS-CoV-2 (Covid-19) virus spread?	All categories
B. OPSUN's health promotion, enunciated as etero-attribution and self-attribution.	5. How could it be possible to intervene in order to promote the health of the healthcare professional who serves cancer patients? (Etero-attribution)	All categories
	9. How would you make interventions or initiatives directed to the promotion of your and your colleagues' health? (Self-attribution)	OPSUN
C. Cancer patient's health promotion.	10. As well as the medical treatments aimed at treating the pathology, how would you increase the health of the cancer patients?	All categories

Note: the answers to the questions have been analysed both singularly, comparing them, and by each of the three areas, observing the different contribution of the respondents' categories for the creation of the investigated configuration.

Table S2. Sample's socio-demographic information.

	n	%
**Age range**		
20-25	16	25.80
26-30	7	11.30
31-35	4	6.50
36-40	3	4.80
41-45	5	8.10
46-50	6	9.70
51-55	11	17.70
56-60	6	9.70
61-65	3	4.80
66-70	1	1.60
**Gender**		
Male	15	24.20
Female	47	75.80
**Total**	**62**	**100**
	**n**	**%**
**Professional role**		
Medical doctor	4	18.20
Nourse	16	72.70
Health and social care worker	2	9.10
**Work experience (oncology)**		
1-5 years	8	36.40
6-10 years	3	13.60
11-20 years	2	9.10
Over 20 years	9	40.90
**Total**	**22**	**100**
